# Evaluation of the prognostic and predictive value of HER family mRNA expression in high-risk early breast cancer: A Hellenic Cooperative Oncology Group (HeCOG) study

**DOI:** 10.1038/sj.bjc.6604769

**Published:** 2008-11-04

**Authors:** A K Koutras, K T Kalogeras, M-A Dimopoulos, R M Wirtz, U Dafni, E Briasoulis, D Pectasides, H Gogas, C Christodoulou, G Aravantinos, G Zografos, E Timotheadou, P Papakostas, H Linardou, E Razis, T Economopoulos, H P Kalofonos, G Fountzilas

**Affiliations:** 1 Division of Oncology, Department of Medicine, University Hospital of Patras, Rion, Greece; 2 Department of Medical Oncology, Papageorgiou Hospital, Aristotle University of Thessaloniki School of Medicine, Thessaloniki, Greece; 3 Hellenic Cooperative Oncology Group, Data Office, Athens, Greece; 4 Department of Clinical Therapeutics, Alexandra Hospital, University of Athens School of Medicine, Athens, Greece; 5 Siemens Healthcare Diagnostics, Cologne, Germany; 6 Laboratory of Biostatistics, University of Athens School of Nursing, Athens, Greece; 7 Department of Medical Oncology, Ioannina University Hospital, Ioannina, Greece; 8 Second Department of Internal Medicine, ‘Attikon’ University Hospital, Athens, Greece; 9 Laiko General Hospital, University of Athens School of Medicine, Athens, Greece; 10 Second Department of Medical Oncology, Metropolitan Hospital, Piraeus, Greece; 11 Third Department of Medical Oncology, Agii Anargiri Cancer Hospital, Athens, Greece; 12 Department of Surgery, University of Athens School of Medicine, Athens, Greece; 13 Oncology Department, Hippokration Hospital, Athens, Greece; 14 First Department of Medical Oncology, Metropolitan Hospital, Piraeus, Greece; 15 First Department of Medical Oncology, Hygeia Hospital, Athens, Greece

**Keywords:** HER family, mRNA, kRT-PCR, prognostic value, predictive value, breast cancer

## Abstract

The aim of the study was to evaluate the prognostic ability of the transcriptional profiling of the HER family genes in early breast cancer, as well as to investigate the predictive value of HER2 mRNA expression for adjuvant treatment with paclitaxel. RNA was extracted from 268 formalin-fixed paraffin-embedded (FFPE) tumour tissue samples of high-risk breast cancer patients enrolled in the randomised HE10/97 trial, evaluating the effect of dose-dense anthracycline-based sequential adjuvant chemotherapy with or without paclitaxel. The mRNA expression of all four HER family members was assessed by kinetic reverse transcription-polymerase chain reaction (kRT–PCR). The overall concordance between kRT–PCR and IHC/FISH for HER2 status determination was 74%. At a median follow-up of 8 years, multivariate analysis showed that EGFR and HER2 mRNA expression was associated with reduced overall survival (OS). HER3 and HER4 mRNA level had a favourable prognostic value in terms of OS and disease-free survival (DFS), respectively. Adjusting for HER2 mRNA expression, OS and DFS did not differ between treatment groups. These data indicate that EGFR as well as HER2 are prognostic factors of worse clinical outcomes, whereas HER3 and HER4 gene transcription is associated with better prognosis in high-risk early breast cancer. However, HER2 mRNA expression did not predict clinical benefit from paclitaxel. Kinetic RT–PCR represents an alternative method for evaluating the expression of HER family members in FFPE breast carcinomas.

Adjuvant chemotherapy improves disease-free survival (DFS) and overall survival (OS) in early-stage breast cancer (EBCTCG, 2005) with taxanes representing active agents in such a treatment. However, chemotherapy is associated with potentially life-threatening side effects. Therefore, the identification of reliable prognostic factors as well as biological markers that might have the ability to predict therapeutic response is crucial. So far, no biomarkers have been identified that can reliably predict clinical benefit from paclitaxel in breast cancer patients.

The human epidermal growth factor receptor (HER) family comprises of four homologous members: ErbB-1 (epidermal growth factor receptor (EGFR) or HER1), ErbB-2 (HER2) for which no ligand has been described so far, ErbB-3 (HER3) which is characterised by its impaired kinase activity, and ErbB-4 (HER4) (Mosesson and Yarden, 2004). There is an extensive literature on the role of the HER family in breast cancer and particularly that of HER2, which is considered to be a key oncogene in breast carcinogenesis. Overexpression or amplification of HER2 occurs in 15–30% of breast carcinomas and is considered to confer a more aggressive biology and an unfavourable impact on the course of the disease (Slamon *et al*, 1987). Moreover, it has been suggested that HER2 overexpression or amplification in breast cancer predicts greater sensitivity to anthracycline-containing chemotherapy (Gennari *et al*, 2008) and resistance to the CMF regimen (Gusterson *et al*, 1992). HER2 may also identify patients who are likely to benefit from higher doses of adjuvant chemotherapy (Dressler *et al*, 2005). The predictive value of HER2 expression regarding the response to taxane-based chemotherapy is considered controversial and the results of the studies have been conflicting, so far (Pritchard *et al*, 2008). Although some trials have supported an interaction between HER2 overexpression and taxane activity (Konecny *et al*, 2004; Hayes *et al*, 2007), others have failed to demonstrate such an association (Gonzalez-Angulo *et al*, 2004). Only a limited number of studies have evaluated the effect of taxane-containing regimens with respect to HER2 status in the adjuvant setting (Martin *et al*, 2005; Kostopoulos *et al*, 2006; Hayes *et al*, 2007).

In the light of clinical data suggesting that HER2 can be useful as a predictive marker for both trastuzumab and chemotherapy, standardised determination of the HER2 status in tumours has become more important. HER2 can be analysed at the DNA, the mRNA or the protein level. Various techniques are available, each with advantages and disadvantages. For practical reasons, immunohistochemistry (IHC) using an anti-HER2 antibody is currently the method of choice for HER2 testing. However, major drawbacks of IHC are that the results are not quantitative, the interpretation is significantly influenced by several technical factors and the inter-observer variation is quite large. Although these discrepancies are improved by the use of standardised IHC tests (such as the HercepTest), it is generally recommended that (2+) HER2 immunostaining needs to be further validated by fluorescence *in situ* hybridisation (FISH) analysis (Ellis *et al*, 2000; Mass *et al*, 2000; Birner *et al*, 2001; Bartlett *et al*, 2003). Despite efforts to standardise these assays, substantial intra- and inter-laboratory variability of the results still exist. Kinetic reverse transcription–polymerase chain reaction (kRT–PCR) has recently been suggested as an alternative technique for the detection and quantification of HER2 expression. kRT–PCR is simple, relatively fast and produces reliable, quantitative and reproducible results. Moreover, it can easily be standardised, reduces inter-observer variability and does not require experienced pathologists for interpretation. However, a disadvantage of this technique is the specific requirements for handling of the tissue specimens to preserve the integrity of RNA. Recent studies have shown that the small amounts of degraded RNA in archival formalin-fixed paraffin-embedded (FFPE) tissues can be successfully amplified and detected using kRT–PCR techniques (Gjerdrum *et al*, 2004).

EGFR is overexpressed in several human tumours and is considered to initiate a variety of important steps during the malignant transformation. It has been estimated that 45% of human breast carcinomas overexpress EGFR (range, 14–91%) (Klijn *et al*, 1992). So far, there are no widely accepted criteria for the determination of EGFR status. EGFR overexpression has been associated with oestrogen receptor (ER) and progesterone receptor (PR) negativity (Pawlowski *et al*, 2000; Ferrero *et al*, 2001; Tsutsui *et al*, 2002; Bieche *et al*, 2003). Moreover, there may be an association between EGFR positivity and high histological grade or lymph node involvement, although not all existing studies are in agreement (Pawlowski *et al*, 2000; Ferrero *et al*, 2001; Witton *et al*, 2003; Rampaul *et al*, 2004; Tzaida *et al*, 2007). Currently, the prognostic significance of EGFR in breast cancer patients remains unclear. In addition, HER3 overexpression has been documented in 20–30% of invasive breast carcinomas. The prognostic significance of HER3 expression is also poorly documented and the available data are conflicting (Quinn *et al*, 1994; Travis *et al*, 1996; Pawlowski *et al*, 2000). With respect to the HER4 receptor, the current evidence suggests that it is characterised by antiproliferative activity (Naresh *et al*, 2006). HER4 overexpression has been reported as a favourable prognostic factor in the literature (Pawlowski *et al*, 2000; Suo *et al*, 2002; Witton *et al*, 2003). The HER family represents therefore an attractive area for the application of targeted therapies in breast cancer and significant treatment advances have been made, so far. As trans-signalling is now considered an essential feature of HER family function, the role of lateral signalling partners is also becoming increasingly important.

In this study, we assessed the prognostic significance of HER family mRNA expression using kRT–PCR, in a series of high-risk early breast cancer patients, treated with dose-dense anthracycline-based sequential adjuvant chemotherapy with or without paclitaxel, within the context of a randomised phase III clinical trial. Furthermore, we investigated whether HER family mRNA expression in the tumour could possibly identify patients who are likely to benefit from the addition of paclitaxel to adjuvant chemotherapy.

## Materials and methods

### Patients

Formalin-fixed paraffin-embedded tissue blocks of primary breast cancer were retrospectively collected from 268 patients who were part of the Hellenic Cooperative Oncology Group (HeCOG) 10/97 trial population. The basic patient and tumour characteristics are shown in Table 1. The HE10/97 trial randomised a total of 595 high-risk (T1-3N1M0 or T3N0M0) breast cancer patients in the period 1997–2000, to receive either 4 cycles of epirubicin (E) followed by 4 cycles of intensified CMF (cyclophosphamide, methotrexate and 5-fluorouracil) combination chemotherapy (E-CMF) or 3 cycles of epirubicin followed by 3 cycles of paclitaxel (T) and 3 cycles of intensified CMF (E-T-CMF). Chemotherapy cycles were administered every 2 weeks and patients received granulocyte-colony stimulating factor (G-CSF) support. The trial was approved from the Bioethics Committee of the Aristotle University of Thessaloniki and patients provided written informed consent prior to enrolment. All participating patients also gave written informed consent for research use of their biologic material. The results of the HE10/97 study have been recently reported (Fountzilas *et al*, 2005).

### Pathologic determinations

Primary tumour diameter and axillary nodal status were obtained from the histopathological report. ER and PR status was assessed by IHC, whereas relative information was provided by the participating institutions according to their own reference laboratories. Tissue paraffin sections stained for ER/PR were considered as positive even when only a small number of neoplastic cells displayed nuclear immunoreactivity. Histological grade was evaluated according to the Scarff, Bloom and Richardson system.

### Molecular and immunohistochemical studies

Owing to the logistical and organisational barriers arising from the retrospective nature of the study, collection of FFPE tumour tissue samples was possible in less than half of the patients enrolled in the HE10/97 prospective clinical trial. RNA was isolated from 268 FFPE tumour tissue samples employing an experimental method based on proprietary magnetic beads from Siemens Healthcare Diagnostics (Cologne, Germany). For all tumour samples included in the analysis the number of malignant cells represented at least 75% of all nucleated cells per section, as verified by haematoxylin–eosin staining. Kinetic RT–PCR was applied for the assessment of the expression of the EGFR, HER2, HER3, and HER4 genes using gene-specific TaqMan™-based primer/probe sets. Forty cycles of nucleic acid amplification were applied and the cycle threshold (*C*_t_) values of the target genes were identified. *C*_t_ values were normalised by subtracting the *C*_t_ value of the housekeeping gene RPL37A from the *C*_t_ value of the target gene (Δ*C*_t_). RNA results were then reported as 40-Δ*C*_t_ values, which would correlate proportionally to the mRNA expression level of the target gene.

In short, each FFPE slide (5 *μ*m thick) was deparaffinised in xylol and ethanol, the pellet was washed with ethanol and dried at 55°C for 10 min. The pellet was then lysed and proteinised overnight at 55°C with shaking. After addition of a binding buffer and the magnetic particles (Siemens Healthcare Diagnostics, Cologne, Germany) nucleic acids were allowed to bind to the particles for 15 min at room temperature. On a magnetic stand, the supernatant was aspirated and the beads were washed several times with a washing buffer. After addition of an elution buffer and incubation for 10 min at 70°C, the supernatant was aspirated on a magnetic stand without touching the beads. After normal DNAse I treatment for 30 min at 37°C and inactivation of DNAse I the RNA quality and quantity was checked by measuring absorbance at 260 and 280 nm. RNA was then used in RT–PCR. The primer/probe sets used for amplification of the target genes were the following:

EGFR Probe CCTTGCCGCAAAGTGTGTAACGGAAT

Forward Primer CGCAAGTGTAAGAAGTGCGAA

Reverse Primer CGTAGCATTTATGGAGAGTGAGTCT

HER2 Probe ACCAGGACCCACCAGAGCGGG

Forward Primer CCAGCCTTCGACAACCTCTATT

Reverse Primer TGCCGTAGGTGTCCCTTTG

HER3 Probe CTCAAAGGTACTCCCTCCTCCCGGG

Forward Primer CGGTTATGTCATGCCAGATACAC

Reverse Primer GAACTGAGACCCACTGAAGAAAGG

HER4 Probe CACAGACTGCTTTGCCTGCATGAATTTC

Forward Primer GAGGCTGCTCAGGACCTAAGG

Reverse Primer GAGTAACACATGCTCCACTGTCATT

Human reference total RNA pooled from 10 human cell lines (Stratagene, La Jolla, California, USA) was used as a positive control. RNA-free DNA extracted from tumour tissues was used as a negative control.

Data regarding EGFR and HER2 protein expression using IHC were available in 241 and 228 patients, respectively (Table 1). EGFR was assessed at the Department of Pathology of the Metaxas Cancer Hospital, Athens, as described earlier (Tzaida *et al*, 2007). HER2 was determined at the Department of Pathology of the Hygeia Hospital, Athens, with additional analysis of cases with an IHC score of 2+ by FISH, as described earlier (Kostopoulos *et al*, 2006).

### Statistical analysis

Categorical data are presented as counts and corresponding percentages, whereas continuous data are presented as medians and ranges. For all receptors, the median was the pre-specified cutoff point and its distinguishing ability for patient prognosis was tested by means of the log-rank test. In case of no distinguishing ability of the median in terms of OS, the plan was to proceed with an exploratory analysis to test if the 25th and 75th percentiles were more appropriate cutoffs. Exploratory analysis was performed in a subgroup of the sample and validated in the rest of the patients. If the 25th and 75th percentiles were not validated as appropriate cutoff points, exploration would continue from the 10th to the 90th percentiles. In case a conclusion was reached on a cutoff point through exploration, subsequent analysis would initially be performed excluding the corresponding gene and repeated including it at the optimal cutoff (ability to distinguish OS significantly in the whole sample). We present the results of the analysis including this gene, only in cases where the results were not altered significantly. Comparison of categorical data between groups of patients was performed using the *χ*^2^-test. Variables included in the comparisons were involved lymph nodes (0–3 *vs* ⩾4), histological grade (good or moderate *vs* poor or undifferentiated), ER and PR status (positive *vs* negative), tumour size (⩽2 *vs* 2–5 *vs* >5 cm), histology (ductal *vs* lobular *vs* other) and age (<50 *vs* >50 years). Continuous data were compared using the Mann–Whitney test, or the Kruskal–Wallis test in case of more than two groups. Correlations among the receptors were assessed using the Spearman's Correlation Coefficient Method.

Overall survival was measured from time of chemotherapy initiation to patient's last contact or death. Disease-free survival was measured from time of chemotherapy initiation to patient's last contact or disease progression. Cases of disease progression, deaths from any cause without verified relapse and second cancers were treated as events in the estimation of DFS (Hudis *et al*, 2007). Survival was estimated using the Kaplan–Meier method. Comparisons between groups of patients, as defined by receptor cutoffs, were performed using the log-rank test. Multivariate Cox analysis including age (<50 *vs* >50 years), involved lymph nodes (0–3 *vs* ⩾4), histology (ductal *vs* lobular *vs* other), histological grade (good or moderate *vs* poor or undifferentiated), size (⩽2 *vs* 2–5 *vs* >5 cm), ER/PR status (positive *vs* negative), hormonotherapy (yes *vs* no), radiotherapy (yes *vs* no), EGFR (⩾75th percentile *vs* <75th percentile), and HER2, HER3, HER4 (⩾median *vs* <median) was performed. Variable selection was performed based on the likelihood ratio test with an exclusion criterion set at 0.10. The final model was adjusted for the group of randomisation (E-T-CMF *vs* E-CMF). Interaction between paclitaxel containing chemotherapy and the genes of interest was also considered. Level of significance was *α*=0.05 for all tests. Results of this study were presented according to reporting recommendations for tumour marker prognostic studies (McShane *et al*, 2005). The statistical analysis was conducted using SPSS 11 for Windows.

## Results

### Normalised mRNA expression of HER family receptors

**Figure 1:**
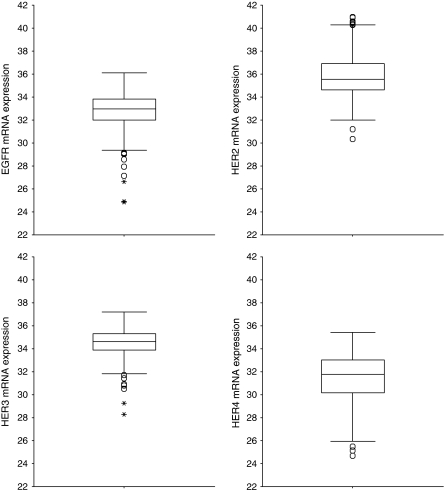
Distribution of breast carcinoma specimens according to normalised expression of mRNA encoding for HER family receptors.

The distribution of tumour samples according to the normalised expression of mRNA encoding for HER family receptors is shown in Figure 1. The median value for EGFR was 32.95 (range, 24.85–36.11), for HER2 35.56 (range, 30.32–40.98), for HER3 34.63 (range, 28.3–37.1), and for HER4 31.79 (range, 24.67–35.43).

### Concordance between kinetic RT–PCR and IHC

The total number of tumours with data available from both IHC and kRT–PCR was 240 and 228 for EGFR and HER2, respectively. For EGFR, 39 of the 240 tumours (16%) were IHC positive, whereas 59 tumours (24.5%) were kRT–PCR positive. For HER2, 64 of the 228 tumours (28%) were IHC/FISH positive, whereas 113 tumours (49.5%) had HER2 mRNA expression above the median, as assessed by kRT–PCR. For these tumours, we found a statistically significant association between the evaluations obtained by the two methods, for the EGFR (Mann–Whitney test, *P*<0.001) and the HER2 (Kruskal–Wallis test, *P*<0.001) receptors. The observed overall concordance between the determination of HER2 by kRT–PCR and IHC/FISH was 74%. The levels for sensitivity and specificity were 92 and 67%, respectively. The overall agreement between kRT–PCR and IHC for EGFR was 75%. Sensitivity and specificity were 49 and 80%, respectively (Table 2).

### Relationships among HER family receptors mRNA expression

A positive correlation was found between HER2 and HER3 mRNA levels (*r*=0.224, *P*<0.001). No association was demonstrated between HER2 and the other two family members. Moreover, HER3 and HER4 mRNA values were positively correlated to each other (*r*=0.444, *P*<0.001) and negatively correlated to EGFR (*r*=−0.143, *P*=0.019 and *r*=−0.125, *P*=0.043, respectively).

### Association of HER family receptors mRNA expression with clinicopathological parameters

EGFR mRNA expression was inversely related to the presence of ER (*P*=0.044). HER2 was positively associated with the number of involved lymph nodes (*P*=0.013). HER3 mRNA expression was associated with ER positivity (*P*=0.017), whereas HER4 was associated with histopathological grade I+II (*P*=0.001) and ER and PR positivity (*P*<0.001 and *P*<0.001, respectively). Furthermore, EGFR mRNA expression was inversely associated with ductal histology (*P*=0.029), whereas that of HER2 was positively associated with ductal histological type (*P*=0.001).

### Prognostic value of HER family receptors mRNA expression

Survival status of the patients was updated in October 2007. The median follow-up time was 95.5 months (95% CI: 92.4–98.6, range, 7–117 months). During this time, 87 patients had developed a relapse and 61 patients had died. The 3-year OS was 93% (95% CI: 90–96%), whereas the 5-year OS was 85% (95% CI: 81–90%). The 3-year DFS was 80% (95% CI: 75–84%), whereas the 5-year DFS was 74% (95% CI: 68–79%).

**Figure 2:**
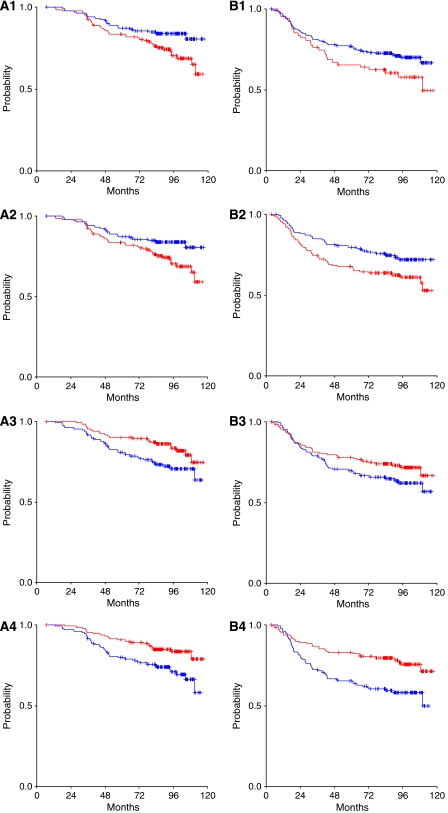
(**A1**) OS (*P*=0.022) and **B1**. DFS (*P*=0.076) for patients with EGFR mRNA expression <75th percentile (*N*=200, blue line) and ⩾75th percentile (*N*=67, red line). (**A2**) OS (*P*=0.024) and **B2**. DFS (*P*=0.026) for patients with HER2 mRNA expression <median (*N*=134, blue line) and ⩾median (*N*=134, red line). (**A3**) OS (*P*=0.026) and **B3**. DFS (*P*=0.135) for patients with HER3 mRNA expression <median (*N*=133, blue line) and ⩾median (*N*=134, red line). (**A4**) OS (*P*=0.010) and **B4**. DFS (*P*=0.001) for patients with HER4 mRNA expression <median (*N*=130, blue line) and ⩾median (*N*=130, red line).

For each of the HER family receptors, three cutoff points (25th, 50th and 75th percentiles) were assessed for prognostic value. In the majority of cases, the median (50th percentile) was the optimal cutoff point. However, in the case of EGFR the 75th percentile was the best threshold, allowing us to distinguish two populations of significantly different prognosis. Using the 75th percentile, patients whose tumours had increased EGFR mRNA expression had significantly reduced OS (22 out of 67 deaths in EGFR-positive *vs* 38 out of 200 deaths in EGFR-negative patients, log-rank *P*=0.022) (Figure 2A1). The hazard ratio (HR) for death in EGFR-positive patients was 1.83 (95% CI: 1.08–3.09, *P*=0.024). The median value was used as a cutoff point for HER2, HER3 and HER4. A significant association between HER2 mRNA overexpression and reduced OS was demonstrated (39 out of 134 deaths in HER2-positive *vs* 22 out of 134 deaths in HER2-negative patients, log-rank *P*=0.024) (Figure 2A2). The HR for death in HER2-positive patients was 1.81 (95% CI: 1.07–3.05, *P*=0.027). In contrast, HER3 as well as HER4 mRNA expression had a favourable prognostic value in terms of OS (HR=0.56, 95% CI: 0.33–0.94, *P*=0.028 and HR=0.50, 95% CI: 0.29–0.86, *P*=0.011, respectively) (Figure 2A3 and A4). Among 134 HER3-positive patients 23 deaths were recorded, whereas among 133 HER3 negative patients 37 deaths were observed (log-rank *P*=0.026). Similarly, among 130 HER4-positive patients 21 deaths were observed *vs* 38 deaths among 130 HER4-negative patients (log-rank *P*=0.010). In multivariate analysis that included 260 patients, EGFR, HER2, HER3, and the number of involved axillary lymph nodes, all independently affected OS (Table 3).

With respect to DFS, elevated HER2 mRNA expression was associated with shorter DFS (52 out of 134 relapses in HER2-positive *vs* 35 out of 134 relapses in HER2-negative cases, log-rank *P*=0.026) (Figure 2B2). The corresponding HR for relapse in HER2-positive patients was 1.62 (95% CI: 1.06–2.49, *P*=0.027). HER4 mRNA expression was associated with lower risk for relapse (HR=0.49, 95% CI: 0.31–0.76, *P*=0.002). Among 130 HER4-positive patients 30 relapses were recorded, whereas 54 relapses were observed in 130 HER4-negative cases (log-rank *P*=0.001) (Figure 2B4). In multivariate analysis (*N*=260), HER4 and the number of involved axillary nodes retained their prognostic significance for DFS (Table 3).

### Prognostic value of HER family members co-expression

Regarding the prognostic significance of specific co-expression patterns of all four HER family receptors, we found that the combination of low EGFR, low HER2, high HER3, and high HER4 mRNA expression was associated with significantly longer OS compared not only with the combination of high EGFR, high HER2, low HER3, and low HER4 mRNA expression (*P*<0.001), but also compared with all other possible co-expression profiles (*P*=0.050). Similar findings were demonstrated for DFS (*P*=0.0021 and *P*=0.031, respectively).

Patients with both EGFR and HER2 elevated expression had significantly worse OS compared with those with either EGFR or HER2 increased mRNA expression (*P*=0.031), but DFS was not significantly worse (*P*=0.164).

### Predictive value of HER family mRNA expression

**Figure 3:**
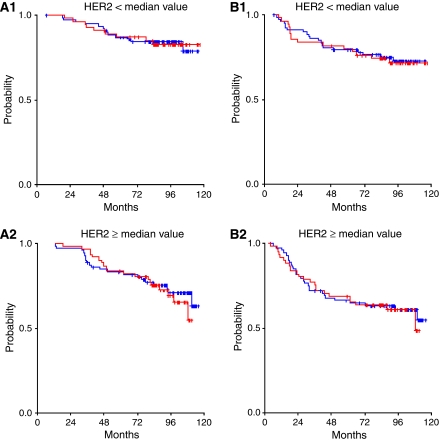
OS (**A1** and **A2**) and DFS (**B1** and **B2**) for patients treated with (red line) or without (blue line) paclitaxel, according to HER2 mRNA expression. **A1** and **B1** (HER2 <median): 55 (41%) E-T-CMF and 79 (59%) E-CMF-treated patients. **A2** and **B2** (HER2⩾median): 62 (46%) E-T-CMF and 72 (54%) E-CMF-treated patients.

The interaction between mRNA expression of HER2 and the addition of paclitaxel was not significant for OS (*P*=0.778). The HR for death in paclitaxel containing chemotherapy (E-T-CMF) among HER2-positive patients was 1.12 (95% CI: 0.60–2.11). With respect to the DFS, the interaction was also non-significant (*P*=0.976). Among HER2-positive cases the HR for recurrence of the paclitaxel containing treatment (E-T-CMF) was 1.05 (95% CI: 0.61–1.81) (Figure 3). In the subgroup of ER-positive patients, the interaction of HER2 mRNA expression and paclitaxel was still non-significant (*P*=0.952 and *P*=0.860 for OS and DFS, respectively). Similarly, the interaction of HER2 mRNA expression and paclitaxel was not found to be significant in the subgroup of ER-negative patients (*P*=0.408 for OS and *P*=0.654 for DFS). In addition, mRNA expression of EGFR, HER3 and HER4 was not predictive for benefit from adjuvant treatment with paclitaxel, neither for OS nor for DFS (data not shown).

## Discussion

In this study, we used kinetic RT–PCR to analyse the transcriptional profiling of the HER family receptor genes, in a comparatively large series of high-risk (predominantly T2–3, node-positive) early breast cancer patients, with a considerably long follow-up of 8 years. Our analysis included gene transcription assessment of all four HER family members. The majority of the clinicopathological studies have focused on protein expression and/or gene amplification of individual HER family receptors. Consequently, the clinical outcome of breast cancer patients with regard to HER family expression as a whole panel remains largely unidentified. In addition, only a small number of trials have evaluated HER family receptors at the mRNA level.

In our patient cohort, the overall concordance between kRT–PCR and IHC/FISH for the determination of HER2 status was good (74%). Our data confirm previous studies demonstrating a substantial agreement between the results of HER2 status evaluation at the mRNA and protein levels (Ginestier *et al*, 2004; Gjerdrum *et al*, 2004; Vinatzer *et al*, 2005). A recent study that compared four different methods of assessment of HER2 status found a good correlation between RT–PCR and IHC, with an overall concordance that varied from 82 to 93% (Ginestier *et al*, 2004). In another study that assessed HER2 status at the DNA, mRNA and protein levels, the concordance of the RT–PCR with the HercepTest was 86.4% (Vinatzer *et al*, 2005). Using the 75th percentile as a threshold in our exploratory analysis, the concordance between the two methods was higher (87%). However, the prognostic ability of HER2 mRNA expression was lost, suggesting that the increase in the cutoff point is likely to miss the effect of lower, but potentially biologically important mRNA levels of HER2. With the use of the median value as a threshold and considering the IHC/FISH as the standard technique for HER2 assessment, the kRT–PCR assay was associated with a high level of sensitivity (92%) and satisfactory specificity (67%). The majority of HER2-positive tumours by IHC/FISH were also categorised as HER2 positive by kRT–PCR (92%). However, among cases showing strong protein expression in IHC, 8% displayed low mRNA expression. This observation may be related either to increased mRNA degradation in FFPE tumour blocks or to accumulation of the protein product, due to aberrant catabolism. Our findings suggest that kRT–PCR is an alternative method for evaluating HER family receptors in FFPE breast tumours. However, routine methods of histological fixation and tissue processing could potentially damage or destroy RNA. In addition, dilution of tumour genomic material by nucleic acids from non-neoplastic tissue components is also a potential source of imprecision (Gjerdrum *et al*, 2004). Furthermore, the required equipment for kRT–PCR is not available in all histopathology laboratories and is quite expensive.

The mRNA expression levels of HER3 and HER4 receptors were positively correlated to each other and negatively correlated to EGFR, in complete agreement with previously reported data (Knowlden *et al*, 1998; Pawlowski *et al*, 2000; Bieche *et al*, 2003). In addition, we demonstrated a positive association between HER2 and HER3 mRNA expression. A similar correlation was described earlier, both at the mRNA and protein level (Bieche *et al*, 2003; Witton *et al*, 2003; Sassen *et al*, 2008). It has been suggested that the HER2/HER3 heterodimer constitutes the most mitogenic dimer in the HER family (Citri *et al*, 2003). HER2 does not bind to phosphatidylinositol 3-kinase (PI3K) and this function is directly mediated through the HER3 receptor (Prigent and Gullick, 1994). With respect to the relationships with the clinicopathological parameters, our findings are in accordance with previous studies evaluating the expression of HER family members in breast cancer, either at the mRNA or the protein level.

In the prognostic analyses, we found a shorter OS in patients with increased EGFR mRNA expression, using the 75th percentile as a cutoff point. In addition, the prognostic significance of EGFR for OS was maintained in the multivariate analysis. EGFR is generally considered to be a negative prognostic factor in breast cancer (Pawlowski *et al*, 2000; Tsutsui *et al*, 2002; Witton *et al*, 2003; Tzaida *et al*, 2007), but up to now, no definitive association between EGFR expression and survival has been demonstrated. The role of EGFR in HER2-mediated cellular transformation is not fully elucidated. Experiments have provided some evidence for a synergistic interaction of these receptors in cellular transformation and induction of mammary tumours (DiGiovanna *et al*, 1998). Moreover, interactions between EGFR and HER2 with respect to the prognosis of breast cancer patients have been reported (DiGiovanna *et al*, 2005). Similarly, in our study, patients with both EGFR and HER2 mRNA overexpression had significantly worse OS when compared to those with either EGFR or HER2 overexpression.

With regard to HER2, we confirmed its negative prognostic significance in terms of OS and DFS. Moreover, HER2 retained its prognostic value for OS in the multivariate analysis. Previous studies investigating the prognostic value of HER2 using real-time RT–PCR showed that this technique is clinically as useful in the assessment of HER2 status as the current standard methods, yielding comparable prognostic information (Vinatzer *et al*, 2005). A recent study (Bergqvist *et al*, 2007) used quantitative real-time PCR (Q-PCR) and RNA expression profiles (RNA-EP) to evaluate HER2 status in relation to clinical outcome in breast cancer patients. Analyses of relapse-free survival and OS on the basis of 5 and 10 years follow-up indicated that, in contrast to IHC/chromogenic *in situ* hybridisation, both Q-PCR and RNA-EP analyses yielded significant results after 10 years of follow-up. These findings suggest that both Q-PCR and RNA-EP are of similar, or even superior, prognostic value compared with the current standard techniques.

The prognostic value of HER3 remains up to now unclear and the available data are contradictory. A number of studies evaluating HER family receptors have indicated a negative prognostic value of HER3 in breast cancer patients (Bieche *et al*, 2003; Witton *et al*, 2003; Sassen *et al*, 2008). In contrast, our present study showed a positive association between HER3 mRNA expression and OS. Moreover, in the multivariate analysis HER3 maintained its prognostic value for OS. Other studies support the positive prognostic ability of HER3 as well (Quinn *et al*, 1994; Pawlowski *et al*, 2000; Lee *et al*, 2007). It has also been shown that a naturally occurring secreted form of the human HER3 receptor is a potent negative regulator of neuregulin-stimulated HER family receptor activation (Lee *et al*, 2001).

Regarding the prognostic significance of HER4, a positive association of HER4 mRNA expression with both OS and DFS was shown. Furthermore, HER4 retained its prognostic power for DFS in the multivariate analysis. Other studies have also supported the favourable prognostic role of HER4 in breast cancer both at the mRNA and the protein level (Pawlowski *et al*, 2000; Suo *et al*, 2002; Witton *et al*, 2003). This positive effect is most likely associated with an inhibitory effect on growth and differentiation signalling. In cell line experiments, when HER2-positive cancer cells were transfected to overexpress HER4, a reduction in proliferation and an increase in apoptosis was observed (Sartor *et al*, 2001). More recent studies have further increased our knowledge regarding HER4-associated apoptosis (Naresh *et al*, 2006).

With respect to the prognostic power of the combined expression profile of all four HER family receptors, we found that the combination of low EGFR, low HER2, high HER3, and high HER4 mRNA expression was associated with a significantly longer OS and DFS, compared to any other combination. This finding suggests that it is the co-expression pattern, rather than the expression of individual family members, that should be taken into account when evaluating the prognosis of the patients and making individualised therapeutic decisions. Moreover, it has been demonstrated that binding of specific ligands to the extracellular domain allows for receptor homo- or heterodimerisation resulting in activation of the cytoplasmatic catalytic function, which leads to receptor autophosphorylation. This autophosphorylation triggers a complex series of signal transduction pathways, such as phosphatidylinositol 3-kinase-Akt, Ras-Raf-MEK-mitogen-activated protein kinase-dependent pathway, PLC–PKC, and JAK/STAT. These pathways affect essential tumorigenic processes, such as proliferation, differentiation, migration, inhibition of apoptosis, and enhanced survival. Therefore, apart from the co-expression of the receptors, the expression of ligands, as well as the cross-talk on different levels among the signal transduction pathways, might also be important.

In this study, we also investigated the predictive ability of the gene transcription of the HER family receptors in tumours of high-risk breast cancer patients. The patients had participated in the randomised HE10/97 trial evaluating the effect of anthracycline-based dose-dense sequential adjuvant chemotherapy, with or without paclitaxel (Fountzilas *et al*, 2005). Moreover, long-term follow-up was available. Patient characteristics were well balanced between the two arms with the exception of grade, a difference also observed in the prospective clinical trial (Fountzilas *et al*, 2005). The unbalance concerning histological grade is an important issue, as it may have an impact on the results. However, since a multivariate analysis was performed, including both grade and randomisation arm, all presented results take into account this unbalance.

To the best of our knowledge, this is the first study evaluating the effect of a taxane-containing regimen *vs* a non-taxane treatment, according to HER2 status at the mRNA level. In the HE10/97 clinical trial, the addition of paclitaxel had no influence in DFS and OS. In our patient cohort, the interaction between HER2 mRNA expression in the tumours and the addition of paclitaxel was not significant. In the entire HE10/97 trial, the hazard of death was significantly reduced when patients with negative hormonal receptor status were treated with paclitaxel. There is evidence that ER positivity may represent a negative predictive factor for the response to chemotherapy in breast cancer (Berry *et al*, 2006). In our patient cohort, performing an exploratory analysis based on ER status, no significant HER2/paclitaxel interaction was found in either ER-positive or ER-negative patients. Therefore, no predictive ability of HER2 mRNA expression for paclitaxel was established in our study.

Recently, investigators from the CALGB 9344 randomised adjuvant trial (Henderson *et al*, 2003) reported that patients with HER2-positive tumours derived significant benefit from the addition of paclitaxel to a doxorubicin–cyclophosphamide regimen regardless of ER status, whereas there was no additional benefit in HER2-negative, ER-positive cases (Hayes *et al*, 2007). In another randomised study comparing docetaxel-based (TAC) with non-docetaxel-containing adjuvant chemotherapy, the observed reduction in the risk for relapse in patients treated with TAC, did not seem to be driven by HER2 status (Martin *et al*, 2005). A recent study, investigating the predictive power of HER2 protein overexpression assessed by IHC in patients who were part of the HE10/97 trial, did not find predictive ability of HER2 for treatment with paclitaxel (Kostopoulos *et al*, 2006). However, a meta-analysis (Dhesy-Thind *et al*, 2008) including the above three trials (Martin *et al*, 2005; Kostopoulos *et al*, 2006; Hayes *et al*, 2007) demonstrated a significant interaction in terms of DFS. Patients with HER2-positive tumours derived greater benefit from the taxane, but there was a significant benefit for both groups. Up to date, clinical results regarding the interaction of HER2 receptor status and the sensitivity to taxanes are contradictory. Furthermore, preclinical data suggest that HER2 overexpression may contribute to paclitaxel resistance in breast cancer cells (Yu *et al*, 1996; Ueno *et al*, 1997). Consequently, cautious interpretation of the available data is required and additional studies are warranted to clarify these relationships.

Regarding EGFR, a recently published study reported that EGFR protein expression, assessed by IHC, was a negative prognostic marker in the absence of paclitaxel in patients with high-risk operable breast cancer (Tzaida *et al*, 2007). In our study, no significant interaction between EGFR mRNA level and treatment with paclitaxel was found for either OS or DFS. Furthermore, no significant interaction of HER3 and HER4 mRNA expression with the treatment group was demonstrated.

In conclusion, the present study suggests that EGFR as well as HER2 mRNA overexpression are prognostic factors of worse clinical outcome in high-risk operable breast cancer patients, whereas HER3 and HER4 mRNA overexpression are both associated with a better prognosis. The combined expression profile of the HER family receptors, and not the isolated expression of individual members, is likely to be more important when assessing the prognosis of the patients. Furthermore, on the basis of our findings, HER2 gene transcription does not predict greater sensitivity to paclitaxel-based adjuvant chemotherapy. In addition, kinetic RT–PCR represents a valid alternative method for detection and quantification of HER family receptor gene expression in FFPE breast tumour tissues.

## Figures and Tables

**Table 1:** Basic patient and tumour characteristics

	**All patients *N*=268**	**E-T-CMF *N*=117**	**E-CMF *N*=151**
*Age (years)*			
Median (range)	51 (22–76)	52 (28–76)	51 (22–76)
			
*Number of nodes removed*			
Median (range)	20 (4–59)	21 (5–59)	20 (4–53)
			
*Number of positive nodes*			
Median (range)	6 (0–54)	7 (0–54)	6 (0–49)
			
	***N* (%)**	***N* (%)**	***N* (%)**
*Positive nodes categories*			
0 nodes	3 (1)	2 (2)	1 (1)
1–3 nodes	58 (22)	22 (19)	36 (24)
4–9 nodes	122 (46)	57 (49)	65 (43)
>9 nodes	85 (32)	36 (31)	49 (32)
			
*Menopausal status*			
Premenopausal	136 (51)	55 (47)	81 (54)
Postmenopausal	132 (49)	62 (53)	70 (46)
			
*Type of operation*			
Modified radical mastectomy	215 (80)	95 (81)	120 (79)
Breast-conserving surgery	53 (20)	22 (19)	31 (21)
			
*Interval from operation*			
<2 weeks	42 (16)	16 (14)	26 (17)
2–4 weeks	126 (47)	64 (55)	62 (41)
>4 weeks	100 (37)	37 (32)	63 (42)
			
*Tumour size*			
⩽2 cm	81 (30)	34 (29)	47 (31)
2–5 cm	136 (51)	62 (53)	74 (49)
>5 cm	51 (19)	21 (18)	30 (20)
			
*Histology*			
Invasive ductal	190 (71)	85 (73)	105 (70)
Invasive lobular	33 (12)	13 (11)	20 (13)
Mixed	30 (11)	12 (10)	18 (12)
Other	10 (4)	4 (3)	6 (4)
Unspecified	2 (1)	1 (1)	1 (1)
Unknown	3 (1)	2 (2)	1 (1)
			
*Grade*			
I–II	135 (50)	48 (41)	87 (58)
III-Undifferentiated	132 (49)	68 (58)	64 (42)
Unknown	1 (0.4)	1 (1)	0 (0)
			
*ER/PR status (IHC)*			
Negative	58 (22)	26 (22)	32 (21)
Positive	206 (77)	89 (76)	117 (77)
Unknown	4 (1)	2 (2)	2 (1)
			
*HER2 overexpression (IHC)*			
No	164 (61)	64 (55)	100 (66)
Yes	64 (24)	30 (26)	34 (23)
Unknown	40 (15)	23 (20)	17 (11)
			
*EGFR overexpression (IHC)*			
No	201 (75)	95 (81)	106 (70)
Yes	40 (15)	15 (13)	25 (17)
Unknown	27 (10)	7 (6)	20 (13)

Patient characteristics are well balanced between the two arms, with the exception of grade (*P*=0.010), a difference also observed in the prospective clinical trial.

**Table 2:** Evaluation of HER2 and EGFR by kRT–PCR compared with IHC

	**HER2 (IHC/FISH) *N*=228**				
	**0, 1+, 2+/FISH(−)**	**2+/FISH(+), 3+**	**NPV/PPV %**	**Sensitivity %**	**Specificity %**
*HER2 (kRT-PCR)*					
Below median	110 (67%)	5 (8%)	96/52	92	67
Above median	54 (33%)	59 (92%)			
					
	**EGFR (IHC) *N*=240**				
	**Negative**	**Positive**			
*EGFR (kRT–PCR)*					
Below 75th percentile	161 (80%)	20 (51%)	89/32	49	80
Above 75th percentile	40 (20%)	19 (49%)			

NPV, negative predictive value; PPV, positive predictive value.

**Table 3:** Multivariate analysis (*N*=260)

	**OS**			**DFS**		
	**HR**	**95% CI**	***P*-value**	**HR**	**95% CI**	***P*-value**
*EGFR*						
<75th percentile	1	—	—	1	—	—
⩾75th percentile	1.71	1.00–2.93	0.050	1.52	0.95–2.44	0.079
						
*HER2*						
<Median	1	—	—			
⩾Median	1.84	1.07–3.17	0.027			
						
*HER3*						
<Median	1	—	—			
⩾Median	0.53	0.30–0.91	0.021			
						
*HER4*						
<Median				1	—	—
⩾Median				0.58	0.36–0.93	0.022
						
*Number of nodes*						
0–3	1	—	—	1	—	—
⩾4	2.42	1.08–5.34	0.032	2.70	1.38–5.28	0.004
						
*Grade*						
I–II				1	—	—
III-Undifferentiated				1.52	0.96–2.41	0.071
						
*Adjuvant hormonotherapy*						
No	1	—	—	1	—	—
Yes	0.48	0.23–0.99	0.50	0.52	0.27–0.99	0.046
						
*Group of randomisation*						
E-T-CMF	1	—	—	1	—	—
E-CMF	0.85	0.51–1.41	0.526	0.93	0.60–1.45	0.928
